# Problem drinking recognition among UK military personnel: prevalence and associations

**DOI:** 10.1007/s00127-022-02306-x

**Published:** 2022-06-04

**Authors:** Panagiotis Spanakis, Rachael Gribble, Sharon A. M. Stevelink, Roberto J. Rona, Nicola T. Fear, Laura Goodwin

**Affiliations:** 1 Mental Health and Addiction Research Group, Department of Health Sciences, University of York, York, UK; 2 School of Psychology, Mediterranean College, Athens, Greece; 3 Department of Psychology, University of Crete, Rethymnon, Greece; 4 King’s Centre for Military Health Research, Department of Psychological Medicine, King’s College London, London, UK; 5 Academic Department of Military Mental Health, Department of Psychological Medicine, King’s College London, London, UK; 6 Liverpool Centre for Alcohol Research, Liverpool Health Partners, Liverpool, UK; 7 Spectrum Centre for Mental Health Research, Division of Health Research, Lancaster University, Lancaster, UK

**Keywords:** Alcohol use disorder, Problem drinking, Problem recognition, UK military, Armed forces

## Abstract

**Purpose:**

Despite the higher prevalence of problem drinking in the UK military compared to the general population, problem recognition appears to be low, and little is known about which groups are more likely to recognise a problem. This study examined prevalence of problem drinking recognition and its associations.

**Methods:**

We analysed data from 6400 regular serving and ex-serving personnel, collected in phase 3 (2014–2016) of the King's Centre for Military Health Research cohort study.

**Measurements:**

Participants provided sociodemographic, military, health and impairment, life experiences, problem drinking, and problem recognition information. Problem drinking was categorised as scores ≥ 16 in the AUDIT questionnaire. Associations with problem recognition were examined with weighted logistic regressions.

**Findings:**

Among personnel meeting criteria for problem drinking, 49% recognised the problem. Recognition was most strongly associated (ORs ≥ 2.50) with experiencing probable PTSD (AOR = 2.86, 95% CI = 1.64–5.07), social impairment due to physical or mental health problems (AOR = 2.69, 95% CI = 1.51–4.79), adverse life events (AOR = 2.84, 95% CI = 1.70–4.75), ever being arrested (AOR = 2.99, CI = 1.43–6.25) and reporting symptoms of alcohol dependence (AOR = 3.68, 95% CI = 2.33–5.82). To a lesser extent, recognition was also statistically significantly associated with experiencing psychosomatic symptoms, feeling less healthy, probable common mental health disorders, and increased scores on the AUDIT.

**Conclusion:**

Half of UK military personnel experiencing problem drinking does not self-report their drinking behaviour as problematic. Greater problem drinking severity, poorer mental or physical health, and negative life experiences facilitate problem recognition.

**Supplementary Information:**

The online version contains supplementary material available at 10.1007/s00127-022-02306-x.

## Introduction

The prevalence of harmful drinking or probable dependence in the UK is 10% among serving and ex-serving personnel [1], higher than the UK general population [2] and military samples from Australia [3], Canada [4], and Germany [5]. Previous UK research suggests that only 14% and 41% of military personnel drinking at harmful and dependent levels, respectively, recognise their problem drinking [6], which is comparable to the general population in England (42% for probable dependence; [2]). The consequences of AUDs (alcohol use disorders) in military personnel are likely to be wide reaching, including a negative impact on mental and physical health [7] and a greater likelihood of having problems with the law and loss of productivity at work [8, 9].

Lack of alcohol-specific problem recognition is a key barrier for seeking treatment, as shown for mental health help seeking among UK ex-serving personnel [10, 11] and US serving personnel [12]. A US study of ex-serving personnel demonstrated that problem recognition, along with positive beliefs about usefulness of psychotherapy, were stronger correlates of mental health services utilization than stigma and external barriers [13]. UK research specific to AUDs also suggests that normalisation of problem drinking can delay help seeking [14].

Results from non-military studies suggest that factors that are associated with higher AUD recognition are greater alcohol consumption [15, 16], greater severity of AUD [17–19], greater physical [16, 17] and mental health problems [17, 19], and recurrent legal problems due to AUD [17] which suggests that problem recognition may become more likely after someone has experienced a negative life event related to their alcohol use. These factors have not been examined among military populations, an environment with its own culture and norms around drinking and health perceptions.

To address this issue, the present study uses data from phase 3 of a large UK military cohort to identify the prevalence of problem drinking recognition among people meeting criteria for problem drinking (defined as harmful or probable dependent drinking), and to examine associations with health and impairment, life experiences, and alcohol use.

## Methods

### Design and sample

Participants were drawn from the third phase of a military cohort study of the health and well-being of the UK Armed Forces, established in 2003 by the King’s Centre for Military Health Research (KCMHR). Sampling and recruitment for this phase are described elsewhere [1].

In brief, phase three took place between October 2014 and December 2016 and included 8,093 respondents (44.3% response rate) [1]. To be eligible, respondents should have participated in either one of the two previous phases and have consented to be contacted again, or they were drawn from a replenishment sample of trained personnel who enlisted after August 2009. Eligible participants were mailed a study information leaflet together with an invitation to complete an online survey. A paper version was also sent if requested, or if there was no reply to the initial invitation.

The sample for this study was restricted to 6400 regular military personnel, full-time reservists, and ex-serving personnel from phase three of the cohort study. We excluded 1688 volunteer reservists (civilians devoting a small amount of time to military duties as part-time soldiers) and five respondents who transferred to the Australian military, as we did not consider them representative of UK military drinking culture and norms.

## Measures

### Socio-demographic and military characteristics

Participants provided information on gender (female/male), marital status (in relationship/not in relationship), whether they had children younger than 18 years old (yes/no), level of education (< A level/ ≥ A level^1^), current or former rank (commissioned officer, non-commissioned officer (NCO), or other), Service branch (Royal Navy, Army, Royal Air Force (RAF)), main role in parent unit (combat vs non-combat, with non-combat including combat support and combat service support roles), and deployment to Iraq or Afghanistan (ever vs never deployed). Peoples' date of birth was provided by the Ministry of Defence and was used to calculate age group at the time of survey completion (< 30, 30–39, 40–49, or ≥ 50).

### Alcohol use disorder

Current alcohol use disorder was assessed with the Alcohol Use Disorder Identification Test (AUDIT; [20]), a 10-item questionnaire with total scores ranging from 0 to 40. AUDIT scores were used to derive AUD severity, drinking patterns, and caseness for problem drinking (defined as meeting criteria for harmful drinking or probable dependence). They were also used in deriving the recognition outcome variable.

AUDIT total scores from all items were used to derive the following levels of severity: (a) hazardous drinking (8–15), (b) harmful drinking (16–19), and (c) probable dependence (AUDIT ≥ 20). Participants falling in the last two categories (16 or above) were categorised as meeting caseness for problem drinking.

The AUDIT sub-domains were used to evaluate drinking patterns. These sub-domains are hazardous alcohol use (including the three items measuring frequency of drinking, typical quantity and frequency of heavy drinking, taking scores 0–12), alcohol-related harm (including the four items measuring guilt after drinking, blackouts, alcohol-related injuries, and other concerned about drinking, taking scores 0–16) and dependence (including the three items measuring impaired control over drinking, increased salience of drinking and morning drinking, taking scores 0–12). Cut-off score were ≥ 10 for hazardous drinking [21] and ≥ 4 for alcohol-related harm and for dependence sub-domains [22]. It should be noted that although levels of AUD severity are mutually exclusive, drinking patterns may overlap; in that, an individual can endorse symptoms of both hazardous drinking and dependence.

Regarding caseness for problem drinking, we linked our data with results from the previous phase of the cohort (phase 2) to derive a measure of previous problem drinking. This was analysed only among participants who participated in both phases.

### Health and impairment

Common mental disorders (CMD) and post-traumatic stress disorder (PTSD) were evaluated with the General Health Questionnaire (GHQ-12); [23] and the National Centre for Post-Traumatic Stress Disorder Checklist-DSM-5 (PCL-5; [24]), respectively. The GHQ-12 measures well-being in the last few weeks using 12 items and a four-point response scale scored as 0–0–1–1. Scores ≥ 4 indicate probable CMD. The PCL-5 measures symptoms of PTSD in the past month using 20 items and a four-point response scale (0-not at all to 4-extremely). Scores ≥ 38 indicate probable PTSD [25].

Somatic symptoms (e.g., stomach pain and back pain) in the past month were assessed using the somatoform disorders scale of the Patient Health Questionnaire (PHQ) [26]. The scale has 15 items (14 for men) and a three-point response scale (0—not bothered at all to 3—bothered a lot). Scores ≥ 10 were categorised as meeting criteria for psychosomatic symptoms.

Respondents completed six items from the SF-36 Health Survey [27]. One item evaluated subjective perceptions of overall health using a five-point response scale (0-poor to 5-excellent) and one item evaluated experiencing social impairment in the past month due to physical or emotional problems using a five-point response scale (0-not at all to 5-extremely). The remaining four items evaluated restrictions in work or other activities in the past month due to health or emotional problem using a binary response scale (yes/no). Responses were used to estimate functional impairment via a binary variable coded as any functional impairment (yes to at least one item) or no functional impairment (no to all items).

### Life experiences

Adverse life events relating to military-related experiences, general adverse life events, and childhood experiences were examined in relation to recognition. To assess military-related experiences, respondents completed a single-item question asking if they experienced any major problems when returning from their most recent Iraq or Afghanistan deployment (yes/no), and a 14-item scale on combat experiences during their most recent deployment (e.g., clear/search buildings, giving aid to wounded, etc.) [28, 29]. Total scores on the scale ranged from 0 to 140, with higher scores indicating greater exposure to combat [30].

General adverse life events in the past 3 years were measured using an adapted version of a 10-item stressful life-events checklist (e.g., divorce or broken relationship) previously used in military populations [31]. Items on physical and mental health problems were excluded as these experiences were covered in other measures. Responses were categorised as 0–1, 2, or ≥ 3 adverse life events. Problems with the law (arrested by the police or charged with criminal offence (yes/no)) were examined as a standalone binary measure.

Measures of family relationship adversities and antisocial behaviours in childhood were adapted from the Adverse Childhood Exposure study scale [32]. We calculated the total number of family relationship adversities reported (e.g., I used to get shouted a lot at home) and categorised responses as 0, 1, or ≥ 2 family relationship adversities [33]. Respondents who reported often getting into physical fights at school, plus at least one additional antisocial behaviour (e.g., playing truant from school), were categorised as demonstrating childhood antisocial behaviour [34].

### Outcome measure of recognition

Respondents were asked "Have you had any alcohol problems in the last three years?" (yes/no), to measure subjective self-identification of an alcohol-related problem. For the main analysis, recognition of problem drinking was defined as meeting criteria for problem drinking (total score on the Alcohol Use Disorder Identification Test ≥ 16) and self-reporting an alcohol problem. However, self-reporting of alcohol problems is also reported here across all levels of AUD severity (hazardous drinking, harmful drinking, or probable dependence).

### Data analysis

All percentages, odds ratios (ORs), and confidence intervals (CIs) were weighted, using sampling and response weights. Sampling weights represented the inverse probability of a respondent being sampled from a specific sub-population and response weights represented the inverse probability of participating, based on factors associated with response (e.g., sex, age, rank, service, or serving status). A single study weight was then calculated by multiplying sampling weights with response weights ([1]). Frequencies were unweighted. The threshold of statistical significance across all analyses was p < 0.05. A complete cases analysis was performed in STATA IC 14 [35].

Factors associated with problem drinking recognition were examined with weighted logistic regressions. Univariable weighted logistic regressions were used to identify sociodemographic and military variables significantly associated with problem drinking recognition, which were then added as covariates in the multivariable models (Online resource 1—Socio-demographic and military variables associated with problem drinking recognition among respondents meeting criteria for problem drinking).

Multivariable weighted logistic regression models examined the associations between key variables of interest (health and impairment, alcohol use disorder, and life experiences) and the outcome of interest (problem drinking recognition). Each variable was tested in a univariable model and then adjusted for the following covariates: (a) age and gender, and (b) education and service status (identified in the sociodemographic univariable models), and (c) caseness for CMD to account for psychological distress.

For post-deployment experiences and past problem drinking, analyses were restricted to personnel who had deployed to Iraq or Afghanistan or had taken part to both phases 2 and 3 of the cohort study, respectively. According to results from the sociodemographic univariable models in these sub-samples, multivariable models were adjusted for a) age, gender, and education, and b) age, gender, and marital status, respectively.

To avoid problems of multicollinearity, the association of probable PTSD with problem drinking recognition was not adjusted for CMD. Harmful drinking patterns (AUDIT sub-domain) were not included as a predictor of problem recognition as almost all respondents who met problem drinking criteria endorsed this pattern of alcohol use (96%).

In a post hoc exploratory analysis, AUDIT scores were added as an additional covariate in the models that examined associations with problem drinking recognition. This was to examine whether greater severity of problem drinking could account for some of the associations (e.g., whether those with poorer health also had more severe problem drinking and therefore were more likely to recognise a problem). For life experiences, we included only adverse life events experienced in the last 3 years, as we did not consider this mechanism to be relevant to the other life experience factors (e.g., childhood experiences).

The analysis presented above was not pre-registered and the results should be considered exploratory.

## Results

### Sample characteristics

Table 1 presents the sociodemographic and military characteristics for the full sample in this study (*N *= 6400) and for respondents meeting criteria for problem drinking (AUDIT ≥ 16, * N * = 602). Both samples were predominantly male, in a relationship, with A level education or higher. Most were serving or had served in the Army in a combat (service) support role as NCOs and had deployed to either Iraq or Afghanistan.

**Table 1:** Socio-demographic and military characteristics of all respondents as well as those meeting criteria for problem drinking (AUDIT ≥ 16)

Variable (valid N)	Full sample (*N* = 6400)			Met criteria for problem drinking (*N* = 602)		
	*n*	%^a^	95% CIs	*n*	%^a^	95% CIs
*Age at survey completion (6400)*						
< 30	1327	13.55	12.74–14.42	154	17.86	14.86–21.31
30–39	2130	35.34	33.90–36.81	219	40.19	35.44–45.13
40–49	1843	32.54	31.15–33.96	159	29.79	25.49–34.48
50 +	1100	18.57	17.48–19.71	70	12.16	9.46–15.50
*Gender (6400)*						
Female	735	9.22	8.47–10.04	42	4.44	3.09–6.35
Male	5665	90.78	89.96–91.53	560	95.56	93.65–96.91
*Marital status (6,263)*						
In relationship	5261	85.72	84.67–86.70	479	81.39	77.39–84.82
Single	1002	14.28	13.30–15.33	121	18.61	15.18–22.62
*Children* < *18yrs (6154)*						
Yes	3165	54.87	53.37–56.35	306	57.50	52.67–62.20
No	2989	45.13	43.64–46.63	284	42.50	37.80–47.33
*Education (6361)*						
None/O levels/GCSEs	1964	32.01	30.63–33.43	226	37.38	32.80–42.20
A level, degree, or higher	4397	67.99	66.57–69.37	375	62.62	57.80–67.20
*Rank (6400)*						
Officer	1654	19.06	18.07–20.09	126	15.79	12.99–19.05
Non-commissioned Officer (NCO)	3711	65.55	64.18–66.89	362	66.10	61.54–70.37
Other	1035	15.39	14.32–16.53	114	18.11	14.65–22.18
*Role in unit (6374)*						
Combat	1642	26.88	25.56–28.24	193	32.58	28.20–37.29
Combat (service) support	4732	73.12	71.76–74.44	408	67.42	62.71–71.80
*Service branch (6400)*						
Royal Navy	1083	17.95	16.82–19.13	96	16.48	13.11–20.52
Army	3854	61.03	59.58–62.46	395	66.84	62.14–71.23
RAF	1463	21.02	19.87–22.22	111	16.67	13.48–20.44
*Serving status (6400)*						
Serving	3712	42.20	40.81–43.60	351	42.04	37.54–46.68
Ex-serving	2688	57.80	56.40–59.19	251	58.96	53.32–62.46
*Ever deployed to Iraq or Afghanistan (6395)*						
Yes	4397	63.45	61.97–64.90	449	70.84	66.09–75.17
No	1998	36.55	35.10–38.03	153	29.16	24.83–33.91

^a^Percentages are out of total valid responses

### Prevalence of recognition

Among the full sample (N = 6400), 10% met criteria for problem drinking, of whom 49% self-reported alcohol problems in the last 3 years.

Table 2 reports the prevalence of self-reported alcohol problems in the past 3 years by AUD severity. Alcohol problems were self-reported by 8% of those at hazardous level (AUDIT 8–15), 33% of those at harmful level (AUDIT 16–19), and 73% of those at a level of probable dependence (AUDIT ≥ 20).

**Table 2:** Prevalence of self-reported alcohol problems split by AUD severity

Risk severity ^b^ (valid N)	"Yes" to "Did you have any alcohol problems in the last three years?"		
	*n*	% ^a^	CIs
Hazardous (*n* = 2398)	168	7.66	6.45–9.08
Harmful (*n* = 368)	115	32.70	27.16–38.78
Probable dependence (*n* = 225)	148	73.40	66.44–79.37

^a^Percentages are row percentages. ^b^Risk-level information (AUDIT score) was provided by 6253 respondents

### Factors associated with problem drinking recognition

Table 3 presents the adjusted ORs and 95% confidence intervals (CIs) for associations between alcohol use disorder factors and problem drinking recognition. Results demonstrated an increase in odds of recognition by one and a half times among respondents with higher total AUDIT scores. There was also an almost twofold and fourfold increase in odds of recognition among respondents meeting criteria for the hazardous drinking pattern domain or alcohol dependence pattern domain, respectively.

**Table 3:** Alcohol use disorder variables associated with problem drinking recognition among respondents meeting criteria for problem drinking (AUDIT ≥ 16) (N = 602)

Variables (valid N)	Problem recognition ^a^		Unadjusted model		Adjusted model 1^b^		Adjusted model 2^c^	
	*n*	%^d^	OR	95% CIs	AOR	CIs	AOR	CIs
AUDIT score (values are mean and SD) (602)	**19.94**	**.22**	1.35**	**1.25–1.45**	1.37**	**1.26–1.48**	1.36**	**1.25–1.47**
Hazardous drinking AUDIT domain (593)^e^								
Non-case	73	38.04	1.00		1.00		1.00	
Case	**190**	**54.67**	1.96**	**1.29–2.99**	1.81**	**1.17–2.83**	1.95**	**1.24–3.08**
Dependence AUDIT domain (593)^f^								
Non-case	145	38.46	1.00		1.00		1.00	
Case	**118**	**69.12**	3.52**	**2.31–5.56**	3.91**	**2.50–6.12**	3.68**	**2.33–5.82**
Past AUD (from phase 2) (389)^g^								
Non-case	90	48.10	1.00		1.00		1.00	
Case	78	50.52	1.10	0.68–1.78	1.14	0.69–1.91	1.15	0.68–1.93

Bold indicates significance

^a^Problem recognition = responding "yes" to "Did you have any alcohol problems in the last 3 years?" ^b^ Adjusted model 1 = Adjusted for age, gender, education, and serving status. ^c^Adjusted model 2 = additionally adjusted for CMD. ^d^Percentages are row percentages and are out of total valid responses. e. Hazardous drinking AUDIT domain cases = domain score ≥ 10. f. Dependence AUDIT domain cases = domain score ≥ 4. g. Restricted to respondents participating in both of phases 2 and 3, adjusted for age, gender, education, and marital status, as these variables were significantly associated with recognition in that sub-sample

*p < .05 ** *p* < .01

Table 4 presents the adjusted ORs and 95% CIs for associations between problem drinking recognition and health and impairment variables. Results demonstrate an almost threefold increase in odds of recognition among respondents who experienced any functional impairment due to health, emotional, or stress problems (vs no impairment), moderate or quite/extreme social impairment due the same reasons (vs no impairment), somatic/physical symptoms (vs no symptoms), or probable PTSD (vs no PTSD). There was also an almost twofold increase in odds of recognition among respondents who met criteria for probable CMD, compared to those who did not. Odds of recognition were lower (almost threefold reduction) among respondents who perceived their overall health as excellent/very good/good (vs poor/fair).

**Table 4:** Health and impairment variables associated with problem drinking recognition among respondents meeting criteria for problem drinking (AUDIT ≥ 16) (*N* = 602)

Variables (valid* N*)	Problem recognition^a^		Unadjusted model		Adjusted model 1^b^		Adjusted model 2^c^	
	*n*	% ^d^	OR	95% CIs	AOR	95% CIs	AOR	95% CIs
Subjective health rating (591)								
Fair/poor	93	67.20	1.00		1.00		1.00	
Excellent/very good/good	**169**	**42.18**	0.36**	**0.22–0.58**	0.37**	**0.23–0.61**	0.44**	**0.27–0.73**
Functional impairment (587)								
No	87	36.08	1.00		1.00		1.00	
Yes	**173**	**58.53**	2.50**	**1.66–3.77**	2.71**	**1.76–4.17**	2.30**	**1.45–3.65**
Probable CMD ^e^ (593)								
Non-case	122	40.72	1.00		1.00		NR ^f^	
Case	**141**	**60.07**	2.19**	**1.46–3.28**	2.16**	**1.42–3.27**	**NR**	**NR**
Probable PTSD ^g^ (590)								
Non-case	196	43.87	1.00		1.00		NR	NR
Case	**66**	**72.14**	3.31**	**1.90–5.79**	2.86**	**1.64–5.07**	**NR**	**NR**
Physical/somatic symptoms (585)								
Non-case	138	38.44	1.00		1.00		1.00	
Case	**118**	**64.28**	2.88**	**1.89–4.40**	2.78**	**1.80–4.30**	2.40**	**1.53–3.78**
Social impairment (590)								
No/slightly	126	37.18	1.00		1.00		1.00	
Moderately	**49**	**63.35**	2.92**	**1.65–5.16**	2.93**	**1.7–5.1**	2.69**	**1.51–4.79**
Quite/extremely	**87**	**66.32**	3.33**	**2.01–5.50**	3.20**	**1.90–5.37**	2.56**	**1.41–4.64**

Bold indicates significance

a. Problem recognition = responding "yes" to "Did you have any alcohol problems in the last three years?". b. Adjusted model 1 = adjusted for age, gender, education, and serving status. c. Adjusted model 2 = additionally adjusted for CMD. d. Percentages are row percentages and are out of total valid responses. e. CMD = common mental disorders. f. NR = Not retained in the second adjusted model, because it was redundant (CMD) or to avoid multicollinearity (PTSD). g. PTSD = post-traumatic stress disorder

^*^ p < .05 ** p < .01

Table 5 presents the adjusted ORs and 95% CIs for associations between problem drinking recognition and life experiences. Results demonstrated an almost threefold increase in odds of recognition among respondents who reported ≥ 3 adverse life events (vs ≤ 1) or ever being arrested by the police/charged with a criminal offence (vs never being arrested). Among respondents who deployed to Iraq or Afghanistan, there was an almost twofold increase in odds of recognition among those who experienced major problems when returning from deployment (vs no problems).

**Table 5:** Life experiences associated with problem drinking recognition among respondents meeting criteria for problem drinking (AUDIT ≥ 16) (N = 602)

Variables (valid N)	Problem recognition^a^		Unadjusted model		Adjusted model 1^b^		Adjusted model 2^c^	
	*n*	% ^d^	OR	95% CIs	AOR	95% CIs	AOR	95% CIs
Adverse life events (563)								
0–1	81	36.36	1.00		1.00		1.00	
2	50	45.12	1.44	0.84–2.45	1.46	0.85–2.50	1.46	0.84–2.54
3 +	**115**	**64.90**	3.24**	**2.01–5.20**	3.23**	**1.96–5.35**	2.84**	**1.70–4.75**
Ever arrested (582)								
No	210	44.93	1.00		1.00		1.00	
Yes	**45**	**74.73**	3.62**	**1.81–7.25**	3.57**	**1.73–7.39**	2.99**	**1.43–6.25**
Family relationship adversities (577)								
0	79	48.34	1.00		1.00		1.00	
1	50	49.95	1.07	0.61–1.85	1.10	0.62–1.94	1.10	0.62–1.95
2 +	130	50.01	1.07	0.68–1.67	1.03	0.65–1.63	0.98	0.61–1.58
Childhood antisocial behaviour (587)								
No	174	46.38	1.00		1.00		1.00	
Yes	87	55.52	1.44	0.94–2.22	1.38	0.88–2.18	1.32	0.83–2.12
Exposure to combat experiences in last deployment (values are mean and SD) (400) ^e^	22.99 (2.01)		1.00	0.99–1.01	1.00	0.99–1.01	1.00	0.99–1.01
Major problems when returning from last deployment (438) ^e^								
No	118	43.40	1.00		1.00		1.00	
Yes	**79**	**62.27**	2.15**	**1.31–3.53**	2.18**	**1.35–3.68**	1.97**	**1.18–3.29**

Bold indicates significance

^a^Problem recognition = Responding "yes" to "Did you have any alcohol problems in the last three years?"

^b^ Adjusted model 1 = Adjusted for age, gender, education, and serving status

^c^Adjusted model 2 = additionally adjusted for CMD.

^d^Percentages are row percentages and are out of total valid responses

^e^Analysis restricted to those who have ever been deployed to Iraq or Afghanistan, adjusted only for age, gender, and education, as serving status was not significantly associated with recognition in that sample

* p < .05 ** p < .01

Online resources 2 and 3 present the adjusted ORs and 95% CI for the post hoc exploratory analysis where AUDIT scores were added as a covariate in the models examining the association of health and impairment factors and life experiences factors with problem drinking recognition. The effects described above were not attenuated to non-significance, indicating that they were not fully accounted by problem drinking severity.

## Discussion

Approximately half of the respondents in this study who met problem drinking criteria (AUDIT ≥ 16) recognised a problem. Recognition was higher among personnel experiencing more mental health problems, impairment, problem drinking severity, and stressors in life.

Problem drinking recognition in our sample was higher compared to findings from the same UK cohort 7 years ago (14% in harmful drinking and 41% in probable dependence, [6]) and the general UK population (42% for probable dependence, [2]). This might be explained by greater use of Alcohol Brief Interventions (ABI) in military contexts. ABI often includes personalised feedback that might facilitate problem recognition (despite doubts as to whether this might lead to changes in drinking behaviours in military studies; [36]). For example, AUD identification and ABI have more recently been delivered on a wider scale in military dental health settings [37]. Additional explanations for the difference with the previous paper [6] include differences in the study samples, with the previous sample restricted to those who had recently deployed, including a smaller number of ex-serving personnel.

Problem drinking recognition was more likely among respondents with more severe problem drinking, as previously found in non-military samples [15, 18, 38], possibly due to these individuals experiencing more alcohol-related negative experiences. For example, 96% of those meeting criteria for problem drinking endorsed items about experiencing guilt after drinking, blackouts, alcohol-related injuries, or others' concerns about their drinking.

Recognition was also more likely among respondents experiencing poorer health and greater impairment, as in non-military studies [16, 17]. These respondents did not necessarily have more severe problem drinking (as suggested in our exploratory analysis), but they might have had problems with their alcohol use for a longer period of time [39] and thus had more opportunities for recognition. It might also be that respondents recognised their problem drinking more easily when they started experiencing negative effects on their health status (although we could not confirm whether the reported health-related problems were necessarily alcohol-related). Problem recognition is a complex process which is likely to be influenced by a range of factors, particularly stigma [40]. For example, protecting oneself from the stigma of adopting a problem drinking identity is easier when one is not experiencing any obvious harms. Furthermore, personal beliefs such as perceived self-control over one's drinking may also influence on problem recognition as self-efficacy is an important factor in alcohol behaviour change [40].

Respondents with problem drinking and mental health comorbidities were more likely to recognise an alcohol problem than those with problem drinking only, as found in non-military studies [17, 19]. Our exploratory analysis showed that this was not due to greater severity of problem drinking among those with mental health comorbidities. Instead, it might be explained by the fact that military personnel are more likely to seek help for a mental health problem rather than problem drinking [41]. Therefore, personnel receiving treatment for their mental health problem may have also had more opportunity for personal reflection and recognition of their problem drinking. Views around treatment seeking may differ in veterans with comorbid problems compared to those with a single problem [14, 42]

Finally, recognition was more likely among respondents who had experienced greater stressors recently in life. These stressors might be the outcome of problem drinking, increasing awareness of alcohol's adverse effects on one's life. For instance, problem drinking may lead to relationship breakdown or legal problems (for example via associations of problem drinking with intimate partner violence; [43, 44]), or financial or employment problems (for example via associations of problem drinking with functional impairment: [7, 9], and loss of productivity: [8]). However, problem drinking might also develop as a coping mechanism to stressful experiences [45]. In this case, problem recognition might become easier as self-blame is reduced, and people attribute their alcohol problems to external, uncontrollable factors (shifting of causal attribution; [46–48]

A strength of the study is the use of a large cohort of UK military personnel, allowing for robust, generalisable estimates. A limitation of the cross-sectional analysis is that, although it is appropriate for assessing the strength of associations, it does not identify unidirectional relationships. Another limitation comes from the time mismatch between the objective measure of problem drinking (AUDIT, referring to the last year) and the subjective measure of self-perceived problems with alcohol (last 3 years). It is possible that some discordant responses may have been, because someone subjectively reported a problem occurring in the last 3 years, but at the time of completing the AUDIT, they were categorised as a lower risk or non-drinker and had cut down or were abstaining due to the previous problem. However, in the main analysis, these participants would not have been included, because it was restricted to those who met current criteria for problem drinking. It is additionally possible that participants with a recent increase in drinking may not have self-reported this due to the wording of the question relating to 3 years. A final limitation was that problem recognition was captured via a binary outcome, which could be less sensitive to change in problem recognition compared to dimensional AUD problem recognition scales.

The main implication of our findings is that greater support might be required to facilitate problem drinking recognition in those drinking at a problematic level but not experiencing current health or social harms from their drinking. To that end, it might be helpful to challenge binary perceptions of alcohol problems (e.g., healthy drinkers vs "alcoholics", [49, 50]) and replace them by a continuum of severity [51, 52]. Going beyond any stereotypical representations of an "alcoholic" (i.e., a person who has lost control over their drinking, experiencing lots of negative consequences) might make people more amendable to alcohol-related information or interventions, before more severe problems develop. We propose that further research is needed to understand beliefs around what point alcohol use becomes a problem in a military context, how this may change when someone leaves services and whether health campaigns and ABI are resulting in changes in stigma and recognition of problem drinking.

## Conclusions

Efforts to facilitate alcohol-problem recognition will have wide reaching benefits given that almost half of those who drink at a harmful or probable dependence level do not recognise their drinking as problematic and those who do not report having additional health and social issues are less likely to perceive their drinking as problematic. It is encouraging that problem recognition in the UK military has increased over a 7-year period, compared to previous studies in the general population.

## Supplementary Information

Below is the link to the electronic supplementary material.

Supplementary file1 (DOCX 20 KB)

Supplementary file2 (DOCX 16 KB)

Supplementary file3 (DOCX 15 KB)

## Data Availability

The data that support the findings of this study are available from King’s Centre for Military Health Research, but restrictions apply. Please contact the King’s Centre for Military Health Research for more information.

## Footnotes

1 A-levels are educational qualifications on specific subjects awarded to students through national exams. Performance in A-levels is an entry criterion for UK Higher Education Institutions.
