# Supplementary material for: Problem drinking recognition among UK military personnel: prevalence and associations
Source: Soc Psychiatry Psychiatr Epidemiol. 2022 Jun 4;58(2):193–203. doi: 10.1007/s00127-022-02306-x (PMC9922231; doi:10.1007/s00127-022-02306-x)
Supplement: Supplementary file 1 — Supplementary file1 (DOCX 20 KB) [file 127_2022_2306_MOESM1_ESM.docx]

**Problem drinking recognition among UK military personnel: Prevalence and associations.**

**Social Psychiatry and Psychiatric Epidemiology**

Panagiotis Spanakis^1,2^, Rachael Gribble^3^, Sharon A.M. Stevelink^3^, Roberto J. Rona^3^, Nicola T. Fear^3,4^ and Laura Goodwin^5,6^

^1^ Mental Health and Addiction Research Group, Department of Health Sciences, University of York, York, UK

^2^ School of Psychology, Mediterranean College, Athens, Greece

^3^ King’s Centre for Military Health Research, Department of Psychological Medicine, King’s College London, London, UK.

^4^ Academic Department of Military Mental Health, Department of Psychological Medicine, King's College London, London, UK.

^5^ Liverpool Centre for Alcohol Research, Liverpool Centre for Alcohol Research, Liverpool Health Partners, Liverpool, UK.

^6.^ Spectrum Centre for Mental Health Research, Division of Health Research, Lancaster University, Lancaster, UK

**Corresponding author:**

Panagiotis Spanakis, panagiotis.spanakis@york.ac.uk

**S1. Socio-demographic and military variables associated with problem drinking recognitions among respondents meeting criteria for problem drinking (AUDIT ≥ 16) (N=602).**

|  | Problem recognition | | Univariable model | | |
| --- | --- | --- | --- | --- | --- |
|  | n | % | OR | 95% CIs |  |
| **Age at survey completion** |  |  |  |  |  |
| <30 | 52 | 40.50 | 0.74 | 0.44 - 1.25 |  |
| 30-39 | 96 | 47.82 | 1.00 |  |  |
| 40-49 | 84 | 57.23 | 1.46 | 0.89 - 2.40 |  |
| 50+ | 31 | 46.42 | 0.95 | 0.51 - 1.76 |  |
| **Gender** |  |  |  |  |  |
| Female | 15 | 31.96 | 0.47 | 0.22 - 1.02 |  |
| Male | 248 | 49.92 | 1.00 |  |  |
| **Marital status** |  |  |  |  |  |
| In relationship | 205 | 46.96 | 0.63 | 0.39 - 1.03 |  |
| Single | 57 | 58.43 | 1.00 |  |  |
| **Children <18yrs** |  |  |  |  |  |
| Yes | 147 | 51.53 | 1.00 |  |  |
| No | 109 | 45.79 | 0.79 | 0.53 - 1.18 |  |
| **Education** |  |  |  |  |  |
| None/O levels/ GCSEs | 114 | 57.24 | 1.00 |  |  |
| A level, degree or higher | 149 | 44.4 | 0.60* | 0.40 - 0.90 |  |
| **Rank** |  |  |  |  |  |
| Officer | 48 | 44.39 | 0.72 | 0.39 - 1.34 |  |
| Non-commissioned  Officer (NCO) | 163 | 49.34 | 0.88 | 0.52 - 1.49 |  |
| Other | 52 | 52.59 | 1.00 |  |  |
| **Role in unit** |  |  |  |  |  |
| Combat | 92 | 55.70 | 1.00 |  |  |
| Combat (service) support | 170 | 45.87 | 0.67 | 0.44 - 1.02 |  |
| **Service branch** |  |  |  |  |  |
| Royal Navy | 41 | 54.49 | 1.27 | 0.74 - 2.18 |  |
| Army | 175 | 48.57 | 1.00 |  |  |
| RAF | 47 | 46.03 | 0.90 | 0.54 - 1.52 |  |
| **Serving status** |  |  |  |  |  |
| Serving | 133 | 42.43 | 1.00 |  |  |
| Ex-serving | 130 | 54.05 | 1.60* | 1.09 - 2.34 |  |
| **Ever deployed to Iraq or Afghanistan** |  |  |  |  |  |
| Yes | 202 | 50.32 | 1.18 | 0.75 - 1.84 |  |
| No | 61 | 46.24 | 1.00 |  |  |

* p < .05 ** p < .01. Problem recognition = Responding "yes" in "Did you have any alcohol problems in the last three years?".
