# Supplementary material for: Problem drinking recognition among UK military personnel: prevalence and associations
Source: Soc Psychiatry Psychiatr Epidemiol. 2022 Jun 4;58(2):193–203. doi: 10.1007/s00127-022-02306-x (PMC9922231; doi:10.1007/s00127-022-02306-x)
Supplement: Supplementary file 2 — Supplementary file2 (DOCX 16 KB) [file 127_2022_2306_MOESM2_ESM.docx]

**Problem drinking recognition among UK military personnel: Prevalence and associations.**

**Social Psychiatry and Psychiatric Epidemiology**

Panagiotis Spanakis^1,2^, Rachael Gribble^3^, Sharon A.M. Stevelink^3^, Roberto J. Rona^3^, Nicola T. Fear^3,4^ and Laura Goodwin^5,6^

^1^ Mental Health and Addiction Research Group, Department of Health Sciences, University of York, York, UK

^2^ School of Psychology, Mediterranean College, Athens, Greece

^3^ King’s Centre for Military Health Research, Department of Psychological Medicine, King’s College London, London, UK.

^4^ Academic Department of Military Mental Health, Department of Psychological Medicine, King's College London, London, UK.

^5^ Liverpool Centre for Alcohol Research, Liverpool Centre for Alcohol Research, Liverpool Health Partners, Liverpool, UK.

^6.^ Spectrum Centre for Mental Health Research, Division of Health Research, Lancaster University, Lancaster, UK

**Corresponding author:**

Panagiotis Spanakis, panagiotis.spanakis@york.ac.uk

**S2. Health and impairment variables associated with problem drinking recognition among respondents meeting criteria for problem drinking (AUDIT ≥ 16) (N=602) (after adding AUDIT scores as a covariate).**

|  | Problem recognition | | Adjusted model | | |
| --- | --- | --- | --- | --- | --- |
|  | n | % | adj. OR | 95% CIs |  |
| **Subjective health rating** |  |  |  |  |  |
| Fair/poor | 93 | 67.20 | 1.00 |  |  |
| Excellent/very  good/good | 169 | 42.18 | 0.43** | 0.25-0.77 |  |
| **Functional impairment** |  |  |  |  |  |
| No | 87 | 36.08 | 1.00 |  |  |
| Yes | 173 | 58.53 | 1.87* | 1.12-3.10 |  |
| **Probable CMD** |  |  |  |  |  |
| Non-case | 122 | 40.72 | 1.00 |  |  |
| Case | 141 | 60.07 | 1.76* | 1.12-2.77 |  |
| **Probable PTSD** |  |  |  |  |  |
| Non-case | 196 | 43.87 | 1.00 |  |  |
| Case | 66 | 72.14 | 1.93* | 1.06-3.52 |  |
| **Physical/Somatic symptoms** |  |  |  |  |  |
| Non-case | 138 | 38.44 | 1.00 |  |  |
| Case | 118 | 64.28 | 2.28** | 1.39-3.73 |  |
| **Social impairment** |  |  |  |  |  |
| No/slightly | 126 | 37.18 | 1.00 |  |  |
| Moderately | 49 | 63.35 | 2.10* | 1.10-4.00 |  |
| Quite/extremely | 87 | 66.32 | 2.00* | 1.10-3.66 |  |

* p < .05 ** p < .01. CMD = common mental disorders. PTSD = post-traumatic stress disorder. Adjusted model = Adjusted for age, gender, education, serving status, CMD and AUDIT score. Problem recognition = Responding "yes" in "Did you have any alcohol problems in the last three years?".
