# Supplementary material for: Problem drinking recognition among UK military personnel: prevalence and associations
Source: Soc Psychiatry Psychiatr Epidemiol. 2022 Jun 4;58(2):193–203. doi: 10.1007/s00127-022-02306-x (PMC9922231; doi:10.1007/s00127-022-02306-x)
Supplement: Supplementary file 3 — Supplementary file3 (DOCX 15 KB) [file 127_2022_2306_MOESM3_ESM.docx]

**Problem drinking recognition among UK military personnel: Prevalence and associations.**

**Social Psychiatry and Psychiatric Epidemiology**

Panagiotis Spanakis^1,2^, Rachael Gribble^3^, Sharon A.M. Stevelink^3^, Roberto J. Rona^3^, Nicola T. Fear^3,4^ and Laura Goodwin^5,6^

^1^ Mental Health and Addiction Research Group, Department of Health Sciences, University of York, York, UK

^2^ School of Psychology, Mediterranean College, Athens, Greece

^3^ King’s Centre for Military Health Research, Department of Psychological Medicine, King’s College London, London, UK.

^4^ Academic Department of Military Mental Health, Department of Psychological Medicine, King's College London, London, UK.

^5^ Liverpool Centre for Alcohol Research, Liverpool Centre for Alcohol Research, Liverpool Health Partners, Liverpool, UK.

^6.^ Spectrum Centre for Mental Health Research, Division of Health Research, Lancaster University, Lancaster, UK

**Corresponding author:**

Panagiotis Spanakis, panagiotis.spanakis@york.ac.uk

**S3. Life experiences associated with problem drinking recognition among respondents meeting criteria for problem drinking (AUDIT ≥ 16) (N=602) (after adding AUDIT scores as a covariate).**

|  |  | | Adjusted model | |
| --- | --- | --- | --- | --- |
|  | n | % | adj. OR | CIs |
| **Adverse life events** |  |  |  |  |
| 0-1 | 81 | 36.36 | 1 |  |
| 2 | 50 | 45.12 | 1.49 | 0.84-2.62 |
| 3+ | 115 | 64.90 | 2.41** | 1.36-4.27 |
| **Ever arrested** |  |  |  |  |
| No | 210 | 44.93 | 1 |  |
| Yes | 45 | 74.73 | 2.50* | 1.09-5.74 |

* p < .05 ** p < .01. Adjusted model = Adjusted for age, gender, education, serving status, CMD and AUDIT score. Problem recognition = Responding "yes" in "Did you have any alcohol problems in the last three years?".
